# In Vivo Assessment of Clobetasol Propionate-Loaded Lecithin-Chitosan Nanoparticles for Skin Delivery

**DOI:** 10.3390/ijms18010032

**Published:** 2016-12-26

**Authors:** Taner Şenyiğit, Fabio Sonvico, Alessandra Rossi, Işıl Tekmen, Patrizia Santi, Paolo Colombo, Sara Nicoli, Özgen Özer

**Affiliations:** 1 Department of Pharmaceutical Technology, Faculty of Pharmacy, Ege University, Bornova, 35100 Izmir, Turkey; ozgen.ozer@ege.edu.tr; 2 Department of Pharmacy, University of Parma, 43124 Parma, Italy; fabio.sonvico@unipr.it (F.S.); alessandra.rossi@unipr.it (A.R.); santipat@unipr.it (P.S.); paolo.colombo@unipr.it (P.C.); sara.nicoli@unipr.it (S.N.); 3 Department of Histology and Embriology, Faculty of Medicine, Dokuz Eylul University, Inciraltı, 35340 Izmir, Turkey; tekmen@deu.edu.tr

**Keywords:** topical glucocorticoids, clobetasol propionate, nanoparticles, anti-inflammatory activity, transepidermal water loss, skin irritation, lecithin, chitosan

## Abstract

The aim of this work was to assess in vivo the anti-inflammatory efficacy and tolerability of clobetasol propionate (CP) loaded lecithin/chitosan nanoparticles incorporated into chitosan gel for topical application (CP 0.005%). As a comparison, a commercial cream (CP 0.05% *w*/*w*), and a sodium deoxycholate gel (CP 0.05% *w*/*w*) were also evaluated. Lecithin/chitosan nanoparticles were prepared by self-assembling of the components obtained by direct injection of soybean lecithin alcoholic solution containing CP into chitosan aqueous solution. Nanoparticles obtained had a particle size around 250 nm, narrow distribution (polydispersity index below 0.2) and positive surface charge, provided by a superficial layer of the cationic polymer. The nanoparticle suspension was then loaded into a chitosan gel, to obtain a final CP concentration of 0.005%. The anti-inflammatory activity was evaluated using carrageenan-induced hind paw edema test on Wistar rats, the effect of formulations on the barrier property of the stratum corneum were determined using transepidermal water loss measurements (TEWL) and histological analysis was performed to evaluate the possible presence of morphological changes. The results obtained indicate that nanoparticle-in-gel formulation produced significantly higher edema inhibition compared to other formulations tested, although it contained ten times less CP. TEWL measurements also revealed that all formulations have no significant disturbance on the barrier function of skin. Furthermore, histological analysis of rat abdominal skin did not show morphological tissue changes nor cell infiltration signs after application of the formulations. Taken together, the present data show that the use of lecithin/chitosan nanoparticles in chitosan gel as a drug carrier significantly improves the risk-benefit ratio as compared with sodium-deoxycholate gel and commercial cream formulations of CP.

## 1. Introduction

Nanomedicine is a promising field that applies nanotechnologies to healthcare with the ultimate aim of developing innovative medicines to improve the diagnosis and treatment of human diseases. Several materials have been proposed to prepare nanomaterials, such as inorganic materials [1,2], lipids [3] and polymers [4]. In the pharmaceutical domain, nanomaterials have found interesting applications in drug delivery as they can modify the fate of the drug in the body, assure control of the release, protect the drug from enzymatic and chemical degradation and improve efficacy and/or reduce toxicity of drugs. Recently, bioinspired, smart and stimuli-responsive nanomaterials have been developed to further boost the efficacy of these materials, allowing specific and targeted delivery to diseased tissues and cells [5,6].

Topical preparations containing corticosteroids are widely used for the treatment of inflammatory skin diseases due to their anti-inflammatory effects. However, repeated or long-term application of topical corticosteroids may lead to numerous adverse events, including atrophy, striae, telangiectasia, purpura, acneiform eruption and perioral rosacea-like dermatitis [7]. One of the options to reduce the adverse effects of topical steroids is to enhance their retention in the skin without augmenting the amount permeated, to reduce the applied dose. Clobetasol-17-propionate (CP) is considered the most potent of the currently available topical corticosteroids, as its vasoconstriction activity is 1800 times higher than hydrocortisone [8], but the incidence of unfavorable side effects is greater than other related compounds [9], limiting its clinical applicability [7]. Some approaches to improve clobetasol administration have been recently described; they include the use of foam [10] or spray formulations [11], the application of ultrasounds [12] and of different kinds of nanocarriers such as microemulsions [13], nanogels [14], nanoparticles, nanocapsules, nanoemulsions [15,16,17] and nanostructured lipid carriers [18,19].

Our group has developed and evaluated in vitro two innovative formulations, a sodium-deoxycholate (Na-DOC) gel containing 0.05% *w*/*w* CP and CP loaded nanoparticles (NP), composed of lecithin and chitosan, dispersed in a chitosan gel. In particular, lecithin-chitosan nanoparticles have been previously proposed as a self-assembled nanometric delivery system for oral [20], topical [21] and nasal delivery [22].

Both the formulations developed showed positive profiles in terms of the skin accumulation of CP in vitro. The Na-DOC gel accumulated a much higher drug amount in the skin with respect to a commercial cream used as reference with the same CP concentration (0.05%) [23]. On the other hand, the CP-loaded NP formulation accumulated the same drug amount as the commercial cream, but starting from a ten-fold lower CP concentration, i.e., 0.005% *w*/*w* [21].

The topical application of nanomedicines is still a topic of much debate, regarding their real benefit over more traditional formulations, their specific mechanism of action in the skin and the safety of potentially biopersistent nanomaterials and their by-products within the skin layers [24]. In the present study, we aimed to evaluate if the in vitro accumulation of clobetasol-17-propionate evidenced with the NP formulation would improve the efficacy in vivo. Furthermore, the skin tolerability of the formulation was investigated and the possible drug release mechanism of the nanoparticles discussed, with the aim to rule out that tissue structure modifications could be the cause of the enhanced efficacy shown when using the nanoformulation.

In order to complement the promising in vitro results, the purpose of this study was to test, in vivo in a suitable efficacy model, the performance of the two innovative formulations, i.e., the Na-DOC gel containing 0.05% *w*/*w* CP and the nanoparticle suspension in chitosan gel (NP) containing 0.005% *w*/*w* CP. A commercial cream containing CP 0.05% *w*/*w* was used for comparison purposes. In vivo pharmacodynamic activity was evaluated using the carrageenan-induced hind paw edema test on rats [25]. The effect of formulations on the barrier properties of the *stratum corneum* was determined using TEWL; histological analysis was performed to evaluate the possible induction of morphological changes.

## 2. Results

### 2.1. Nanoparticle Characterization

The clobetasol propionate-loaded nanoparticles were characterized before inclusion in the chitosan gel. The average particle size obtained by dynamic light scattering (DLS) was 248 ± 15 nm with a narrow nearly monodisperse distribution (Polidispersity Index 0.1105), while the ζ potential measured by phase analysis light scattering (PALS) had a positive value, i.e., +34 ± 2 mV, in agreement with previous data [21]. Indeed, the structure of the particles has been investigated previously: chitosan and lecithin interact electrostatically to form essentially a multilayered structure [26,27]. These particles are able to load lipophilic drugs in the phospholipid bilayers and the loading can be improved by adding pharmaceutical oils such as triglycerides or fatty acid esters. In fact, isopropyl myristate (IPM) affects the structure of the multilayer chitosan/lecithin nanoparticles by intercalating the phospholipid bilayers, allowing the loading of a much higher quantity of corticosteroid [28].

**Figure 1:**
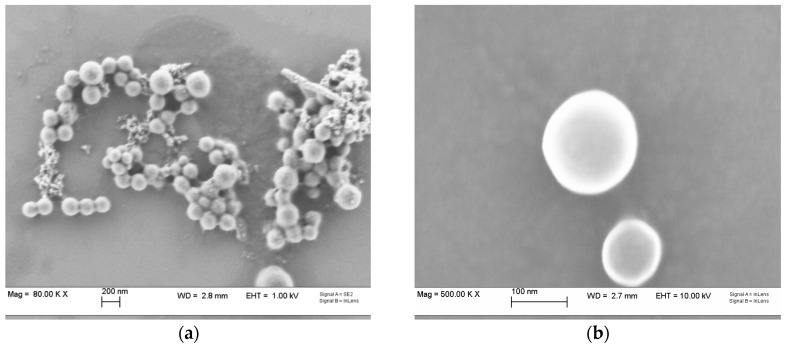
Scanning electron microscopy (SEM) images of the chitosan/lecithin nanoparticles: at 80 K× (**a**) and 500 K× (**b**) magnification.

To further confirm the presence of a rigid shell, a scanning electron microscopy (SEM) was performed. The images obtained (Figure 1) show particles with a diameter of 100–150 nm. Particles were almost perfectly spherical and despite not having being metal-coated, no sign of melting or damage from the electron beam was visible, suggesting a rigid shell surface. The discrepancy between the size results obtained by SEM and DLS could be ascribed to the fact that DLS measures a hydrodynamic diameter, based on the NP diffusion coefficient, and thus it also takes into account the hydration layer surrounding the particles [29].

### 2.2. Anti-Inflammatory Activity Studies

The anti-inflammatory effect of CP-loaded NP and Na-DOC Gel after topical administration was evaluated using carrageenan-induced acute edema in a rat paw model [25]. This model is well-established and has been previously used by numerous authors to evaluate the anti-inflammatory [30,31] and anesthetic [32] properties of topically applied semisolid formulations.

**Figure 2:**
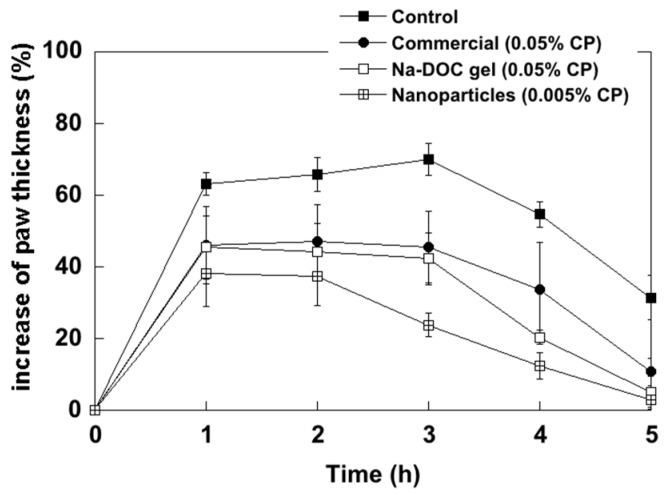
Increase of paw thickness (%) as a function of the time after carrageenan injection.

The results obtained are illustrated in Figure 2, which shows the percent increase in paw thickness as a function of time after carrageenan injection. The data obtained with Na-DOC gel and with the nanoparticles have been compared to the ones obtained using a reference commercial cream (Dermovate^®^, GSK, Uxbridge, UK).

The increase in paw thickness was significantly lower (*p* < 0.05) than for the control group at all time points for each and every formulation tested. However, while the commercial formulation and Na-DOC gel had similar efficacy, the NP formulation performed significantly (*p* < 0.05) better at the third and fourth hour. It is worth mentioning that this higher activity is obtained despite a 10-fold lower drug concentration in the nanoparticle-containing formulation, supporting the evidence that the formulation is more relevant than the drug concentration in the process of CP absorption [7].

From Figure 2, the areas under the curve (*AUC*) were calculated and the values obtained are reported in Table 1 together with the percentage of edema inhibition calculated according to Equation (1).

The table includes also in vitro data obtained in previous papers using isolated pig skin [21,23]. These in vitro data, obtained after 6 h of application, were characterized by:

- A 10-fold higher CP accumulation in the epidermis starting from the Na-DOC gel compared to NP and commercial cream;
- A comparable CP level in the dermis for the three formulations (see the very high data variability).

It appears that no clear correlation exists between in vitro epidermal drug levels (highest skin retention is observed for Na-DOC gel) and in vivo efficacy (highest edema inhibition is observed for NP). Furthermore, the comparable CP levels found in the dermis for the 3 formulations cannot explain the superior in vivo performance of the NP-containing formulation.

However, considering the distribution of CP between epidermis and dermis, commercial cream and NP-containing gel are characterized by deeper skin accumulation. The percentages accumulated in the dermis, in fact, are 8% for NP and 12% for cream, but less than 2% in the case of NA-DOC gel.

### 2.3. Evaluation of Skin Barrier Function and Damage

Skin barrier properties and possible skin irritation and skin damage induced by the formulation applied can be assessed by transepidermal water loss (TEWL) measurements [33] and histology. In fact, it has been shown that, in rat skin, TEWL increase is directly correlated to barrier function impairment produced by irritants. In particular, sodium lauryl sulfate exposure increased TEWL approximately 8 times compared to the control value (10.3 g·m^−2^·h^−1^) [34]. Typical basal values of TEWL obtained in the present work were in the order 11.1–12.6 g·m^−2^·h^−1^, close to the literature data reported before. The TEWL value of rat skin is typically higher than the value of human skin (5–6 g·m^−2^·h^−1^), probably due to the high density of hair follicles in rat skin [35].

**Figure 3:**
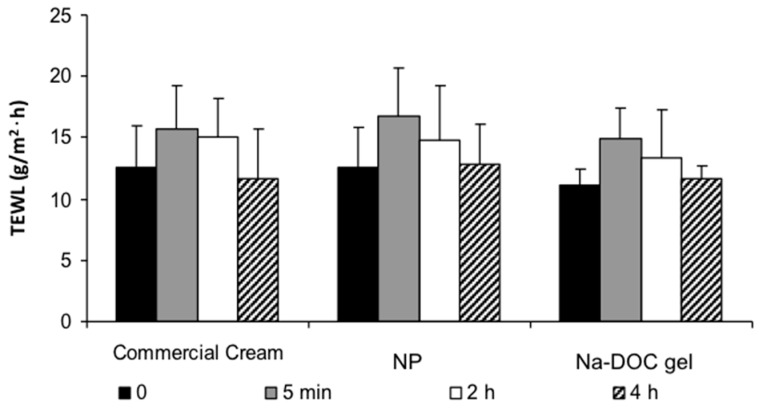
Transepidermal water loss (TEWL) values recorded before formulation application and 5 min, 2 and 4 h after its removal. The effect on TEWL of Dermovate (commercial cream), of the CP-loaded nanoparticles formulation (NP) and of a sodium deoxycholate gel (Na-DOC gel) were compared. All products were applied for 1 h.

Figure 3 shows the variation of TEWL before the application of the formulations and 5 min, 2 and 4 h after its removal (application time was 1 h). It is well known that corticosteroids increase the TEWL value as a signal of the disturbance of skin barrier function [36]. However, for all tested formulations TEWL did increase in a non-significant way when measured 5 min post-application, then decreased gradually to reach the basal value after 4 h. The reason for the increase of TEWL after formulation removal is probably the evaporation of residues of water contained in the formulation. Overall, at least for the application time examined in this study, the commercial cream, NP and Na-DOC gel formulation produced no significant disturbance on the barrier function of *stratum corneum* after a single application.

**Figure 4:**
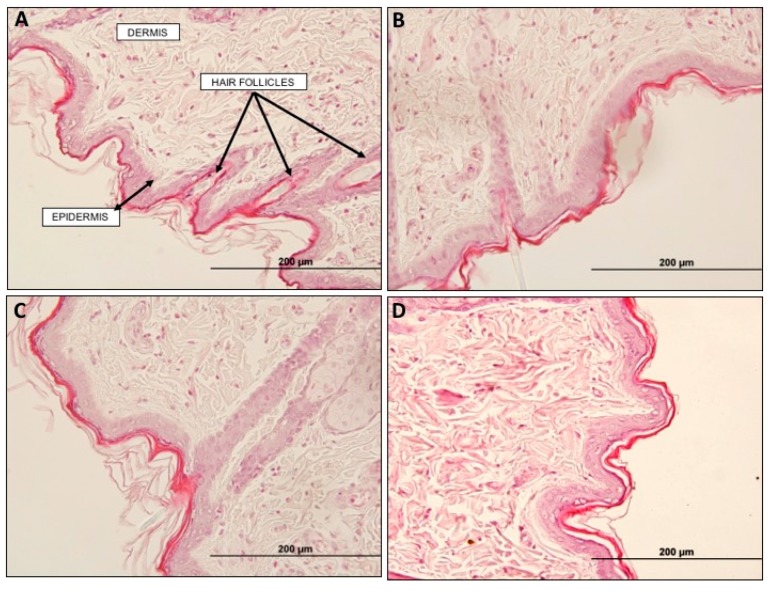
Histology of the rat skin samples. (**A**) Control; (**B**) Commercial cream; (**C**) NP formulation; (**D**) Na-DOC gel.

Skin morphological changes, such as epidermal liquefaction, edema of collagen fibers and cell infiltration can be used as parameters to evaluate the possible damaging effects of the formulation. However, as already suggested by transepidermal water loss (TEWL) data, histological analysis of the rat skin did not reveal morphological tissue changes, at least after a single application of the formulations in this study. No cell infiltration signs were observed, and the structures of the stratum corneum, epidermis and dermis were preserved (Figure 4).

## 3. Discussion

The use of nanoparticles in topical formulations is a topic of debate, especially concerning their safety and their ability to penetrate skin layers [37]. In this study, the potential of nanoparticles for the improvement of the topical administration of glucocorticoids was demonstrated by the higher efficacy shown by a gel containing clobetasol propionate-loaded chitosan/lecithin nanoparticles in comparison to a conventional formulation and a commercial cream. In the carrageenan-induced hind paw edema test in rats, the nanoparticle formulation outperformed the other preparations despite containing a drug concentration ten times lower. Edema development after carrageenan injection has been described as a biphasic event, where the initial phase is mainly histamine- and serotonin-mediated, while prostaglandin-like substances are responsible for the second phase that is thus more sensitive to anti-inflammatory drugs [38].

Interestingly, the differences between the nanoparticle-containing formulation and the other formulations were evidenced in this second phase, as shown in Figure 2 where differences between the formulations appear more significant after 2 h. This may suggest a higher and faster availability of CP from the NP formulation at the site of action that, in this case, is represented by the edema where the inflammatory cells are mainly located.

However, this result appears not to be directly correlated to the in vitro accumulation of the drug in skin layers. Data reported in previous studies, in fact, indicate that the sodium deoxycholate gel is the formulation that provides the highest accumulation in the most superficial skin layers (Table 1). Reasonably, the high CP amount found in epidermis using Na-DOC gel is mainly located in the *stratum corneum*, and thus not available to elicit anti-inflammatory activity on the edema. This is plausible, considering the high lipophilicity of this molecule, characterized by a logP of 3.98 ± 0.61 (ACD/I-Labs, version 15.01, Advanced Chemistry Development, Inc., Toronto, ON, Canada, www.acdlabs.com, 2015). At the same time, the comparable CP levels found in the dermis for the three formulations (Table 1) cannot explain the superior in vivo performance evidenced by the formulation containing the chitosan/lecithin nanoparticles. However, these differences could be attributed to the fact that the in vitro situation is probably different from the in vivo situation, when inflammation and edema are present. In this case, in fact, the augmented capillary permeability can cause a higher CP absorption into the systemic circulation, resulting in a faster clearance from the inflammation site. In this context, nanoparticles can have a positive effect since they can accumulate inside the hair follicles, forming a drug reservoir less susceptible of fast drainage and clearance. Indeed, follicular accumulation of topically applied nanoparticles has been reported by many authors [39,40]. Follicular accumulation of NP can take place in the hind paw model used here, since, contrarily to human palms and plants, which are hairless, Wistar rat foot pads contain hair follicles, although with a much lower density than normal rat skin [41].

In addition, recently, the boost of tamoxifen intestinal permeation after encapsulation in chitosan/lecithin nanoparticles and in presence of specific enzymes such as lysozyme and lipases has been highlighted [20,42]. Lysozyme in particular is an antibacterial protein found in many physiological secretions, able to degrade chitosan and hence to biodegrade the chitosan/lecithin nanoparticle structure. Interestingly, lysozyme is among skin-produced antimicrobial peptides, and pilosebaceous follicle cells, hair bulb cells and eccrine sweat glands have been found to be positive for lysozyme [43,44]. This suggests that nanoparticle accumulation in the skin annexes could contribute to explain the outstanding performance of the nanoparticle formulation containing clobetasol propionate in two ways:

- By forming a reservoir of the drug;
- Through a progressive enzyme-controlled delivery of the drug from the nanometric delivery system.

Considering these two aspects, the formulation containing the nanoparticles appears to provide an optimal timing for the delivery of the drug and is probably the reason why the efficacy of this formulation is more than 10 times that of a traditional gel or commercial cream.

## 4. Materials and Methods

### 4.1. Materials

Clobetasol-17-propionate (CP) was a kind gift from GlaxoSmithKline (Ankara, Turkey). Lecithin (Lipoid S45) was purchased from Lipoid AG (Ludwigshafen, Germany) and chitosan (Chitoclear FG, specifications: deacetylation degree 95%, viscosity 93 cP for 1% *w*/*v* solution in 1% *v*/*v* acetic acid) for nanoparticles preparation was from Primex (Haugesund, Norway). Medium molecular weight chitosan (specifications: MW 190-310 kDa, deacetylation degree 75%–85%, viscosity 200–800 cP for 1% *w*/*v* solution in 1% *v*/*v* acetic acid) for gel preparation as well as λ carrageenan used for hind paw test was obtained from Sigma-Aldrich (St. Louis, MO, USA). Polyethylene glycol 400 (PEG 400) and mannitol were provided from Merck (Darmstadt, Germany). Sodium deoxycholate (Na-DOC) was purchased from Fluka (Munich, Germany). All other chemicals were of analytical grade.

A commercial drug product in form of cream containing 0.05% of clobetasol-17-propionate, Dermovate^®^ (GSK, Uxbridge, UK) was used as reference formulation.

### 4.2. Nanoparticle Preparation and Characterization

Lecithin/chitosan nanoparticles were prepared following the method previously reported by Sonvico and coworkers [45] by direct injection of soybean lecithin alcoholic solution into chitosan aqueous solution.

Briefly, an ethanol solution of lecithin 2.5% (*w*/*v*) containing 0.625% *w*/*v* of CP and 2% *w*/*v* of isopropyl myristate (IPM) was prepared. Nanoparticles were obtained by rapidly injecting 4 mL of the lecithin ethanol solution through a glass pipette (internal diameter 0.75 mm, injection rate 40 mL/min) under mechanic stirring (Ultraturrax TP 18/10-10N, IKA Werke, Staufen, Germany), into 46 mL of a chitosan solution obtained by diluting with distilled water 0.5 mL of 1% (*w*/*v*) chitosan solution in 0.275 N HCl. In the colloidal suspension, lecithin/chitosan ratio was 20:1 (*w*/*w*) and the final CP content was 0.050% *w*/*v*.

Nanoparticles were characterized for size, polydispersity index (PDI) and surface charge using dynamic light scattering (DLS) and phase analysis light scattering (PALS) (ZetaPALS, Brookhaven, Holtsville, NY, USA).

In the case of particle size measurements, samples were diluted in ultrapure 0.45 µm filtered water in order to obtain an average count rate below 150 kcps to avoid multiple scattering. Measurements were performed at 25 °C allowing the sample to equilibrate for 1 min and acquiring six measurements of 60 s each for each sample. The average size and PDI were expressed as mean and standard deviation of the measures obtained for three different batches.

For surface charge measurements, samples were measured directly without dilution, allowing the instrument to automatically optimize signal intensity of the sample. Measurements were performed at 25 °C, collecting scattered light at 15° and repeated 10 times for each sample. The instrument software, applying Smoluchowski approximation, calculated the ζ potential of samples. The ζ potential values were expressed as mean and standard deviation of the measures obtained for three different batches.

Particles morphology was evaluated by scanning electron microscopy (SEM). The sample was diluted 1:10 in ultrapure water (Purelab Flex, ELGA-Veolia LabWater, Zoppola, Italy) and 20 µL were deposed on an aluminium stub and left to dry. The sample was then observed without further metallization using an Ultra High Resolution Field Emission SEM (SUPRA 40, CarlZeiss, Oberkochen, Germany) operated at an operating voltage of 1.00 kV.

### 4.3. Preparation of Na-DOC Gel

The composition and method of preparation of the sodium deoxycholate gel was taken from a previous work published by Valenta and collaborators [46]. In particular, 0.5% sodium deoxycholate (Na-DOC) was dissolved in phosphate buffer saline (0.1 M phosphate buffer pH 7.2 containing 0.9% sodium chloride) and 5% of mannitol was added. Finally, 0.05% (*w*/*w*) CP dissolved in 10% (*w*/*w*) PEG-400 was added to Na-DOC Gel with continuous stirring until uniform distribution.

### 4.4. Preparation of NP Loaded Chitosan Gel

A chitosan gel in water was prepared by dissolving 2 g of chitosan in 100 mL of acetic acid 1.5% *w*/*v* aqueous solution. Then, the colloidal suspension of CP-loaded nanoparticles was incorporated in the chitosan gel in 1:9 (*w*/*w*) ratio to obtain a final CP concentration of 0.005% *w*/*w*.

### 4.5. In Vivo Studies

Male Albino Wistar rats, weighing 180–220 g, were used. They were housed in standard environmental conditions and fed with standard rodent diet with water ad libitum. The experimental protocol was approved by the Local Animal Ethical Committee of Ege University, Faculty of Pharmacy (Approval No. 2007/12-1).

#### 4.5.1. Anti-Inflammatory Activity Studies

To determine the anti-inflammatory activity of the formulations prepared, carrageenan-induced hind paw edema test was performed [25,47]. Twenty rats were divided into four groups, one being the untreated control. Commercial cream, Na-DOC Gel and nanoparticle colloidal suspension incorporated in chitosan gel (NP) were applied to the plantar surface of the rat hind paw at a fixed CP dose of 0.125 mg/cm^2^ for one hour, before the induction of inflammation. In order to produce inflammation, 50 µL of a 1% (*w*/*v*) solution of λ carrageenan in saline was injected into the plantar side of the right hind paw of rats. The contralateral paw received the same volume of sterile saline. The paw thickness (indication of edema) was measured using digital micrometer (Mitutoyo Corp., Kawasaki, Japan) before carrageenan injection and then after 1, 2, 3, 4 and 5 h. The % of increase in the paw thickness was calculated as the difference between the values obtained in right and left paw expressed as percentage. The obtained values were reported as a function of time after carrageenan injection; from the graph, AUC was calculated using Kaleidagraph software (Version 4.01, Synergy Software, Reading, PA, USA). The percentage of inhibition of carrageenan-induced paw edema was then calculated according to the Equation (1) presented above:

(1) $$\%\mathrm{Inibition}=\frac{AUC_{C}-AUC_{f}}{AUC_{C}}\times 100$$

where *AUC_C_* is the average area under the curve of the control rats (no treatment) and *AUC_f_* is the area under the curve of the rats treated with the different formulations.

#### 4.5.2. Transepidermal Water Loss (TEWL)

Before TEWL measurements, rats were anesthetized by intraperitoneal injection of ketamine (30 mg/kg). After recording pre-treatment TEWL values, 0.5 g of formulation (Na-DOC Gel, nanoparticle colloidal suspension incorporated in chitosan Gel or commercial cream) was spread uniformly on 2 × 2 cm area of shaven rat abdomen (4 cm^2^) and left for 1 h. Transepidermal water loss (TEWL) measurements were obtained a Tewameter TM 300 (Courage + Khazaka electronic GmbH, Köln, Germany) applied on the skin for 1 min. TEWL values were recorded 5 min, 2 and 4 h after the removal of the formulation applied on the skin by means of a dry cotton pad. The laboratory temperature and relative humidity were kept constant in the range of 24–25 °C and 45%–50% RH, respectively.

#### 4.5.3. Histological Analysis

Twenty-four hours after treatment, animals were humanely euthanized under anesthesia (urethane 1 g/kg and α-chloralose 50 mg/kg). In order to evaluate the histology of the skin at the site of application, the skin was dissected and fixed with buffered 10% formalin solution for 48 h. Tissue samples were embedded in paraffin following a routine protocol. Subsequently, sections 5 µm thick were stained with hematoxylin and eosin (H&E). The slides were examined using a light microscopy (Olympus BH-2, Tokyo, Japan) and the histopathological appearance of tissues in the different groups were compared. Possible structure changes and cell infiltration were evaluated by two analyzers blinded to the treatments.

### 2.6. Statistical Analysis

Each experiment was replicated at least five times and the values reported are expressed as mean ± standard deviation. Statistical differences were determined using ANOVA test. Significance was determined by Bonferroni test as a post hoc test. Data were considered significant at *p* < 0.05.

## 5. Conclusions

In this study, clobetasol propionate-loaded lecithin/chitosan nanoparticles in chitosan gel demonstrated a significantly higher anti-inflammatory activity compared to a sodium deoxycholate gel and commercial cream (Dermovate) containing the same drug. This result was obtained despite the 10-fold lower (0.005% vs. 0.05%) drug concentration in the nanoparticle-enabled formulation. This result can be explained by considering the well-known capability of nanoparticles to accumulate inside the hair follicles, thus forming a reservoir that slowly releases the drug. In this latter case, the biodegradability of the nanoparticles could be the basis of a further capability of these nanocarriers to promote absorption through the skin. In fact, as a consequence of their specific composition, i.e., polysaccharides and lipids, lecithin/chitosan nanoparticles are most likely biodegraded by enzymes present in skin secretions, such as lysozyme.

Concerning the formulation tolerability, according to the results of TEWL all tested formulations did not significantly disturb the skin barrier. Furthermore, histological analysis of rat abdominal skin did not show morphological tissue changes or cell infiltration signs after application of the formulations.

Finally, lecithin/chitosan nanoparticle-in-gel formulation showed high skin tolerability with significantly improved efficacy over a classic gel formulation and marketed product. This, along with the use of a ten times lower dose, may represent remarkable progress to reduce the dose-dependent side effects and to increase the risk-benefit ratio of clobetasol propionate.

## Figures and Tables

**Table 1:** Comparison between in vivo anti-inflammatory efficacy data (AUC and % of inhibition) obtained after formulations application and in vitro CP accumulation (µg/mg) after application in isolated pig skin [21,23].

Formulation	CP Loading (% *w*/*w*)	In Vivo Efficacy		In Vitro CP Skin Accumulation	
		AUC_0–5_ (%·h) ^1^	Edema Inhibition (%) ^2^	Epidermis (µg/mg)	Dermis (µg/mg)
Dermovate	0.05	178 ± 44	34 ± 16	0.03 ± 0.03	0.0042 ± 0.0027
Na-DOC Gel	0.05	151 ± 23	44 ± 9	0.67 ± 0.19	0.0115 ± 0.0043
NP	0.005	113 ± 16	58 ± 6	0.06 ± 0.03	0.0053 ± 0.0012

^1^ AUC_0–5_ of the control is 269 ± 12; ^2^ Calculated using Equation (1).
